# The Impact of Leadership Styles on Quality and Financial Performance in High Medicaid Nursing Homes

**DOI:** 10.1093/geroni/igaa057.070

**Published:** 2020-12-16

**Authors:** Justin Lord, Ganisher Davlyatov, Akbar Ghiasi, Robert Weech-Maldonado

**Affiliations:** 1 LSU-Shreveport, Shreveport, Louisiana, United States; 2 University of Alabama at Birmingham, Birmingham, Alabama, United States; 3 University of Incarnate Word, San Antonio, Texas, United States

## Abstract

This study examines the association between leadership styles on resident quality and financial performance in under resourced nursing homes (70% or higher Medicaid census). The Bonoma/Slevin leadership model was used to classify managers into four categories, autocrat, consultative autocrat, consensus manager, and shareholder manager. Survey data from 391 nursing home directors (response rate of 37%) from 2017- 2018, were merged with secondary data from LTCFocus, Area Health Resource File, Medicare Cost Reports, and Nursing Home Compare. Two models were ran to examine the effect of leadership styles on the dependent variable(s) nursing home STAR data (quality) and operating margin (financial performance). The independent variables were composite scores for leadership styles, with autocrat as the reference group. Control variables included organizational (ownership, chain affiliation, size, occupancy, payer mix, staffing, and race/ethnicity), and county factors (Medicare Advantage penetration, per capita income, poverty, education, unemployment, and competition). Multivariate regression was used to model the relationship between leadership styles and nursing home quality and financial performance. The consultative autocrat was associated with lower quality (p < 0.05), while the consensus manager was associated with lower profit margin (p < 0.05), as compared to autocratic leadership. The consultative autocrat, who solicits information from the staff yet still makes all significant decisions, is associated in lower quality; however, a consensus manager, who delegates their authority to the group, is associated with lower financial performance. Under-resourced nursing homes who face dual pressures need to recognize trade-offs of different decision making styles for quality and financial performance.

